# Occupational exposure and human carriage of *Streptococcus equi* subsp. *zooepidemicus* resulting in multiple livestock outbreaks

**DOI:** 10.1016/j.onehlt.2025.101063

**Published:** 2025-05-06

**Authors:** Matheus de O. Costa, Richard Rusk, LeeAnn Peters

**Affiliations:** a Large Animal Clinical Sciences, Western College of Veterinary Medicine, University of Saskatchewan, Canada; b Population Health Sciences, Faculty of Veterinary Medicine, Utrecht University, the Netherlands; c Department of Emergency Medicine, Max Rady College of Medicine, Rady Faculty of Health Sciences, University of Manitoba, Winnipeg, Manitoba, Canada; d Maple Leaf Foods Incorporated, Manitoba, Canada

**Keywords:** Streptococcus, Zooepidemicus, Amphixenosis, Zoonosis, Human, Pig, Swine, Infection, Outbreak, Epidemiology, Asymptomatic, Subclinical

## Abstract

**Background:**

*Streptococcus equi* susbsp. *Zooepidemicus* is a cause of septcaemia and an occupational hazard. It was previously thought to only infect humans, and no evidence is available that humans can become long-term carriers of this pathogen.

**Methods:**

Over 3 years two different, both naïve, independent swine farms experienced outbreaks of *S. zooepidemicus.* Site A was depopulated three times, and had three outbreaks. Site B only had one outbreak. Isolates were genetically profiled through whole genome sequencing. Potential carriers and environmental load were tested by a strain-specific real time PCR.

**Findings:**

Environmental samples and non-human carriers tested negative throughout outbreaks. Isolates recovered from pigs in all outbreaks from site A and site B had >99.9 % average nucleotide identity. Phylogenetic analyses suggested that all isolates are related. Patient 1, who transited between site A and B immediately before site B experienced their first swine case, was present in site A after removal off all pigs and before each outbreak. They also shed *S. zooepidemicus* on their mask and were positive by real time PCR from nasopharyngeal swabs.

**Interpretation:**

A human carrier of *S. zooepidemicus* was likely colonized during the first outbreak in pigs on site A. They shed the agent which resulted in multiple outbreaks in Site A, and introduction of the pathogen to Site B. This is the first recorded case of amphixenosis due to *S. zooepidemicus*, evidencing that humans can become colonized and spread the agent to animals.

**Funding:**

Natural Sciences and Engineering Research Council, Canada. Saskatchewan Agriculture Ministry, Canada. Results Driven Agriculture Research, Canada.

## 1 Introduction

*Streptococcus equi* subspecies *zooepidemicus* (Szoo) is reported as a zoonosis linked to animal exposure (*e.g.* handling of livestock or household pets), and consumption of inappropriately cooked animal products. Sepsis, meningitis, pneumonia, arthritis and myositis have been identified as a result of Szoo infection [[1], [2], [3], [4], [5], [6]]. A review of human cases found that incubation period ranged from 1 to 21 days (median 7 days), fever was reported in the majority of the cases, as well as meningeal signs as 75 % (15/20) of the cases had bacteremia. Mortality rate was 30 % (6/20), and at least 45 % of the patients were previously healthy [7]. Szoo is considered an emerging zoonosis and is often portrayed as a challenge to human health under a One Health lens, mostly due to its broad animal host range.

Szoo is described as an opportunistic pathogen of several warm-blooded hosts, including equine, canine, feline, and swine. It is part of the indigenous microbiota of healthy horses, found on skin and mucus membranes of the respiratory and reproductive tracts. It can cause severe disease characterized by pneumonia, septicemia and meningitis in animals [[8], [9], [10], [11], [12], [13], [14], [15]]. Similarly to other β-haemolytic streptococci, Szoo is often susceptible to penicillin and it is considered the drug of choice to treat infections in humans and companion animals [7,9]. Szoo genetic diversity is rich, when compared to other streptococci, which helps explain its promiscuous lifestyle and wide range of hosts [3,16]. A multilocus sequence typing (MLST) scheme for Szoo was developed in 2008, which allowed the comparison of clinical and subclinical isolates to the sequence types (ST) deposited in the online (PubMLST, https://pubmlst.org/organisms/streptococcus-zooepidemicus) [17]. To date, there are 553 STs described. This tool has been key in epidemiological investigations of Szoo outbreaks, given that a serotyping scheme has not yet been developed for this pathogen.

In April 2019, the first case of fatal septicemia due to Szoo infection in pigs was reported in North America [11]. Outbreaks were associated with high mortality rates of adult female animals, often reported as sudden death with no clinical signs. Abortion rates soared up to 11× higher than expected. Attempts to control the disease resulted in multiple failed efforts - herd-wide use of autogenous vaccines, and antimicrobials did not prevent new cases. Resolution could only be achieved through removing animals from the premises, complete sanitation of the barns followed by repopulation with pigs negative for Szoo (termed depopulation).

Here we describe the first confirmed case of Szoo amphixenosis in a swine barn staff – where humans and animals posed a threat to animals and other humans as biological vectors of Szoo. This raises several concerns, including public health, livestock biosecurity and health and companion animal health.

## 2 Methods

Signed, written informed consent was obtained from all study participants prior to the commencement of this investigation. Research ethics approval was waived in light of public health concerns.

### 2.1 Outbreak 1 – site A index case

In March 2019, a highly biosecure commercial swine operation (site A) experienced a high-mortality outbreak of septicaemic bacteremia due to Szoo [11]. This site houses 3000 sows. Patient 1, who was part of the staff in this site since 2005, participated in the removal of approximately 95 % of the dead stock during the outbreak, which lasted 11 months. As the outbreak continued into the summer, Patient 1 reportedly executed their tasks without the use of proper personal protective equipment due to the elevated temperatures inside the barn. At that time, nasopharyngeal swabs were collected from the staff (including Patient 1) and submitted for culture only. Szoo was not identified in any samples. By March 2020, all animals were removed from Site A and the premises and equipment were washed and disinfected. Repopulation with naïve pigs was attempted in May 2020.

### 2.2 Outbreak 1 – site B

Site B is a commercial farm that houses 1200 sows. It is located 60 km away from site A. In November 2019, Patient 1 was moved from site A to site B to address staff shortages. By January 2020, this site also experienced a Szoo outbreak, and the barn was decommissioned by May 2020.

### 2.3 Outbreak 2 – site A

In June 2020, Patient 1 returned to site A. By December 2020, this site experienced a second outbreak of Szoo-associated disease. In March 2022, animals were removed from the premises, followed by another cycle of complete washing and disinfection of the building and any equipment used that could not be replaced. It was repopulated in June 2022.

### 2.4 Outbreak 3 – site A

**Fig. 1:**
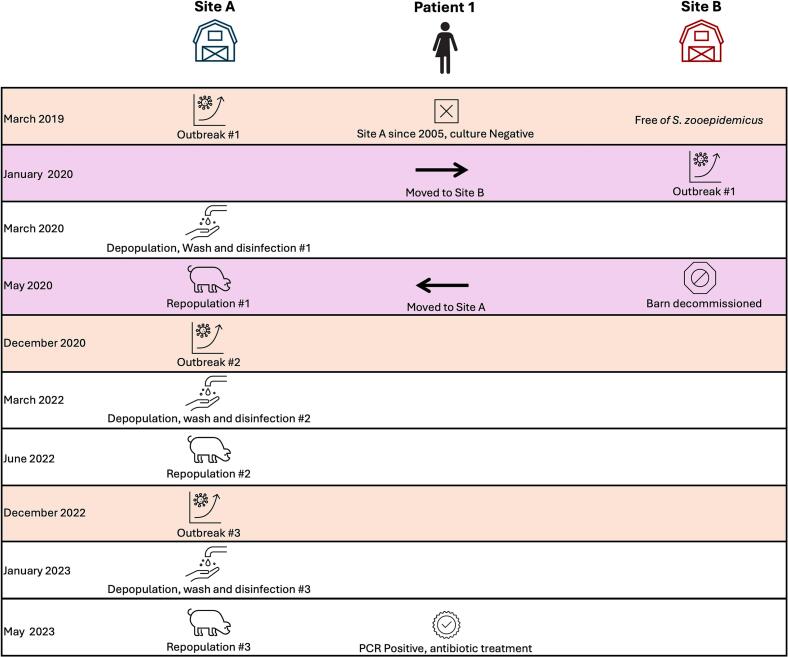
Events associated with Patient 1 movement and outbreaks in pigs, in chronological order. Orange shaded events represent outbreaks, and pink shaded events represent movement by Patient 1. (For interpretation of the references to colour in this figure legend, the reader is referred to the web version of this article.)

In December 2022, Site A experienced a third Szoo outbreak. Patient 1 was still working at this site. Animals were removed from the premises in January 2023, followed complete washing and disinfection of the premises, including any equipment that could not be removed and replaced, and repopulated in May 2023. Nasopharyngeal swabs from the staff on this site were tested in March 2023 using real-time PCR. Until October 2024, Site A did not experience any more outbreaks associated with Szoo. A summary of these events is shown in Fig. 1.

### 2.5 Microbiological diagnostics

A clinical laboratory, fully accredited by the Canadian Association for Laboratory Accreditation, used a standard operation procedure and standard culture media for culturing nasopharyngeal swabs. Briefly, swabs were plated in sheep blood agar plates and incubated at 37 °C in a 5 % CO_2_ atmosphere. For identification, β-haemolytic isolates were applied to a matrix-assisted laser desorption-ionization time-of-flight mass spectrometry (MALDI-TOF, Biotyper, Bruker Daltonics, East Milton, ON, Canada) in a routine thin layer, overlaid with 1.5 μl of matrix solution and analysed.

Szoo and Szoo sequence type 194-specific real-time PCR was performed as previously described [18]. Briefly, DNA from samples was extracted using the MagMax Core Nucleic Acid extraction kit (Applied Byosystems, ThermoFisher Scientific, Ottawa, ON). PCR reactions were carried using a AgPath-ID One-step RT-PCR (Applied Byosystems, ThermoFisher Scientific, Ottawa, ON), and all reaction plates included a no template, blank and bacterial positive control. Samples were analysed in duplicates, and cycling conditions included 120 s at 95 °C, followed by 40 cycles of 5 s at 95 °C and 33 s at 60 °C. Duplicates with a Ct variation greater than 1 were re-analysed. A sample was considered positive if Ct <35, and suspectedly positive if Ct <40. This test was performed at a laboratory fully accredited by the American Association of Veterinary Diagnostic Laboratories (ISO 17025).

### 2.6 Respirator sampling

Nanofibre N95 (3 M, Maplewood, MN, USA) respirators with valves were voluntarily donated from staff following a minimum use time of 1 h in the barn. Respirators were individually bagged, and transported overnight to the laboratory under refrigeration. Upon arrival, masks were handled in a laminar flow cabinet using sterile instruments. The front piece (or valve) was removed, and placed in DNA extraction buffer, according to the manufacturer's guidelines for extraction from swabs.

### 2.7 Environmental sampling

Following barn depopulation (#2, June 2022), washing and disinfection from site A, environmental samples were collected from: gestation pens concrete walls, gestation hallway walls and floors, loadout ramp, farrowing doors and walls, water source, manure pits, attic insulation, gestation crate walls, windows, insects, electronic sow feeder panel, metal posts inside pens, light gaps, ventilation inlets, and feedline tubes. All samples were transported overnight, refrigerated, and submitted for real-time PCR testing.

### 2.8 Treatment and colonization evaluation

Patient 1, the only positive for Szoo, was treated with 300 mg of phenoxymethylpenicillin TID orally for 21 days. Following treatment, nasal and pharyngeal swabs from the same patient were collected and tested monthly for three months using the real-time PCR test described above.

### 2.9 Whole genome sequencing and analyses

Genomic DNA was extracted from Szoo isolates obtained from diseased pigs found dead or euthanized (Table 1) using the DNeasy ultraclean microbial kit (QIAGEN, Toronto, Ontario, Canada). DNA libraries were prepared using a Rapid Sequencing Kit (SQK-RAD0003, Oxford Nanopore Technologies, Oxford, UK). Libraries were sequenced on a GridION sequencer (Oxford Nanopore Technologies, Oxford, UK) using a Flow Cell model FLO-MIN 106 (v R9.4) running the software MinKNOWN v1.10 (Oxford Nanopore Technologies, Oxford, UK) with default parameters. Genomes were trimmed, assembled, annotated and analysed using the comprehensive genomic analysis tool in Pathosystem Resource Integration Center (PATRIC) [19], including phylogenetic analysis using the codon-tree method using 500 randomly chosen coding sequences and 100 rounds of bootstrapping. Multi-locus sequence typing was performed *in silico* based on the PubMLST scheme (http://pubmlst.org). Average nucleotide identity was calculated using JSpecies [20].

**Table 1:** Swine *Streptcoccus equi* subsp. *zooepidemicus* isolates recovered during outbreaks.

Isolate	Outbreak date	Sample of origin	Sequente Type	Site	Farm status
166,442	March 2019	Spleen	ST-194	A	Before depopulation/First outbreak
166,439	March 2019	Spleen	ST-194	A	Before depopulation/First outbreak
2,034,079	March 2020	Lung	ST-194	B	New introduction
20–17,394	December 2020	Liver	ST-194	A	After depopulation/Second outbreak
2,300,608–1	January 2023	Spleen	ST-194	A	After depopulation/Third outbreak
2,300,608–2	January 2023	Lymph node	ST-194	A	After depopulation/Third outbreak

## 3 Results

All staff in Site A, including Patient 1, were sampled using nasal and pharyngeal swabs and tested negative for Szoo based on culture in March 2020 (*n* = 27). Staff working on Site A (*n* = 12) in January 2023 also had nasopharyngeal swabs collected, all of which did not yield Szoo in culture. In February 2023, an alternate approach was used by sampling N95 ventilators (n = 12) and testing using a ST-194-specific PCR. The N95 ventilator sample from Patient 1 was the only positive sample, with a Ct = 37.27, which was considered suspect positive. Confirmation was performed using a nasopharyngeal swab tested by ST-194 specific real-time PCR only, yielding a Ct = 25.28. Following treatment (penicillin V 300 mg TID for 21 days), Patient 1 tested negative by real-time PCR three consecutive times.

**Fig. 2:**
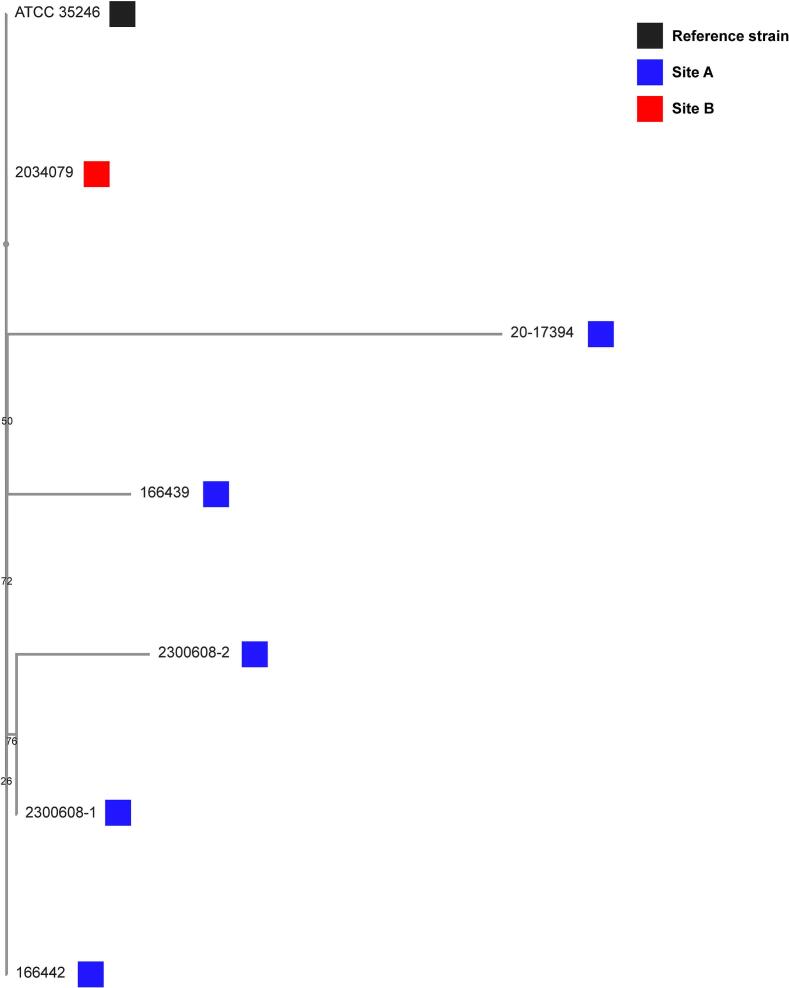
Phylogenetic (codon) tree based on 500 randomly selected coding regions from swine *Streptococcus equi* subsp. *zooepidemicus* isolates obtained from site A (blue block) or site B (red block). ATCC 35246 strain (black blocks) was included for reference. Branch numbers reflect bootstrapping confidence. (For interpretation of the references to colour in this figure legend, the reader is referred to the web version of this article.)

Szoo isolates obtained from pigs during all outbreaks were typed as ST-194, including the index case in March 2019 and subsequential outbreaks until 2023, at different sites (Table 1). Average nucleotide identity between all swine isolates was >99.9 % (Table 2). Phylogenetic analyses revealed that all isolates are related to the reference strain initially isolated from a pig in China, in 1970s, and minimal diversity was detected in the 2023 re-break in Site A (Fig. 2).

**Table 2:** – Average nucleotide identity (%) between different isolates and the reference strain isolated from a diseased pig in China, 1977 (ATCC 35246). Numbers between brackets indicate the percentage of aligned nucleotides.

	2,300,608–1	2,300,608–2	20–17,394	166,442	166,439	ATCC 35246	2,034,079
**2,300,608–1**	*	99.96 [100.00]	99.95 [100.00]	99.94 [99.15]	99.94 [97.28]	99.91 [99.43]	99.95 [99.2]
**2,300,608–2**	99.96 [100.00]	*	99.96 [100.00]	99.95 [99.15]	99.94 [97.29]	99.92 [99.43]	99.96 [99.3]
**20–17,394**	99.95 [100.00]	99.96 [100.00]	*	99.95 [99.15]	99.94 [97.28]	99.92 [99.43]	99.94 [99.7]
**166,442**	99.93 [99.34]	99.94 [99.34]	99.93 [99.34]	*	99.95 [97.53]	99.94 [98.77]	99.93 [98.74]
**166,439**	99.94 [99.13]	99.94 [99.13]	99.94 [99.13]	99.96 [99.36]	*	99.93 [98.56]	99.91 [99.51]
**ATCC 35246**	99.92 [99.99]	99.93 [99.99]	99.92 [99.99]	99.93 [99.16]	99.93 [97.29]	*	99.91 [99.67]
**2,034,079**	99.95 [99.2]	99.96 [99.3]	99.94 [99.7]	99.93 [98.74]	99.91 [99.51]	99.91 [99.67]	*

All environmental samples tested negative for Szoo and Szoo ST-194 through real-time PCR.

## 4 Discussion

Szoo has been known to cause zoonotic infections, either through the ingestion of raw or poorly cooked food or direct exposure to diseased animals [[1], [2], [3],^6^,^16^]. We report here the first documented case of amphixenosis, where a human, as biological vector, was initially infected through exposure to diseased pigs and their secretions. As this subject moved to different, naïve swine barns, it was responsible for introducing Szoo to these herds and triggering economically important outbreaks. This subject was likely a healthy carrier for at least 3 years. To the best of our knowledge, there are no cases reported in the literature presenting evidence where humans were infected by exposure to animals, became asymptomatic carriers of Szoo and disseminated the agent back to animals.

We were able to genetically profile isolates from diseased pigs across the two different sites, site A before (2019) and after two depopulation cycles (2020 and 2023), and site B as a new introduction (2020). All isolates were typed as ST-194, and nucleotide identity was >99.9 %. They are most dissimilar to the ATCC 35246 reference strain. This suggests that the same strain was responsible for the different outbreaks. ST-194 has been isolated in other outbreaks in pigs, as well as in humans who ingested raw pork [2].

While environmental contamination cannot be completely ruled out as the source of the outbreaks on Site A following the index case this is highly unlikely as no environmental sample tested positive for Szoo ST-194. This bacterium is known to survive poorly in the environment, even in the presence of biological material. Prior research found that survival is limited to 5 days at 20 °C, and to a maximum of 15 days at 37 °C [21,22]. During the outbreaks described here, premises were thoroughly washed and disinfected, and remained free of pigs for at least 60 days. As part of a depopulation procedure, vermin are also removed from the site. The only animals with full access to the building during the depopulation period were humans, and sites were repopulated with pigs known to be negative for Szoo ST-194.

At the time of the index case in 2019, Szoo diagnosis in humans and animals relied on aerobic culture from throat or nasal swabs. As a highly contaminated sample, healthy subjects yield negative culture for Szoo, resulting in no isolate obtained from Patient 1 in that instance. There are no reports in the literature describing the prevalence or isolation of Szoo from healthy humans. While we cannot rule out that Patient 1 carried Szoo prior to 2019, that individual worked in the same barn, in direct contact with pigs, for over 15 years. It is expected that, if they were carriers prior to 2019, cases of septicaemic disease due to Szoo ST-194 would have been identified. Clearance of the carrier state was only achieved after antibiotic treatment for 21 days, and confirmed using real-time PCR.

In summary, we described a case of amphixenosis associated with *Streptococcus equi* subsp. *zooepidemicus* associated with humans and pigs. In this instance, the agent was maintained in humans following transmission from pigs, whom then transmitted it to naïve pigs at a different location. This finding has severe implications for the swine industry, in terms of biosecurity and disease control. It also impacts any other livestock industry, as well as those working with wildlife, as Szoo is capable of colonizing a wide range of hosts. Finally, physicians should be aware that humans can be asymptomatic carriers, treatment plans should be designed accordingly, and PCR should be used in favour of culture in cases where the agent is suspected but culture fails to detect it.

## Data sharing

Data used in the preparation of this paper and presented in figures, and tables are available from the corresponding author from the date of publication of this paper, upon reasonable request and pending agreement from relevant ethics committees (for clinical data) and the completion of a written agreement. Any data provided will be de-identified. Genome sequences are available from the date of publication of this paper at the Bacterial and Viral Bioinformatics Resource Center repository (bv-brc.org).

## CRediT authorship contribution statement

**Matheus de O. Costa:** Writing – review & editing, Writing – original draft, Visualization, Validation, Supervision, Software, Resources, Project administration, Methodology, Investigation, Funding acquisition, Formal analysis, Data curation, Conceptualization. **Richard Rusk:** Writing – review & editing, Methodology, Investigation. **LeeAnn Peters:** Writing – review & editing, Funding acquisition.

## Role of the funding source

Funding sources had no role or involvement in this study.

## Funding statement

No specific funding was provided for this investigation. National Sciences and Engineering Research Council - Alliance Program ALLRP 561369-2020 funded the development of the real time PCR technique used here.

## Declaration of competing interest

The authors declare that they have no known competing financial interests or personal relationships that could have appeared to influence the work reported in this paper.

## Data Availability

The data that support the findings of this study are available on reasonable request from the corresponding author, MOC.
